# Genomic, phenotypic and demographic characterization of *Mycobacterium tuberculosis* in Israel in 2021

**DOI:** 10.3389/fcimb.2023.1196904

**Published:** 2023-10-18

**Authors:** Yelena Losev, Mor Rubinstein, Israel Nissan, Paz Haviv, Yohi Barsky, Martha Volinsky, Gefen Bar-Giora, Tamara Zouher, Mazal Hamawi, Gal Zizelski Valenci, Ina Kutikov, Hasia Kaidar Shwartz, Zeev Dveyrin, Daniel Chemtob, Efrat Rorman

**Affiliations:** ^1^ National Public Health Laboratory, Public Health Directorate, Ministry of Health, Tel Aviv, Israel; ^2^ Department of Tuberculosis (TB) and AIDS and National TB Program Manager, Ministry of Health, Jerusalem, Israel; ^3^ Hebrew University-Hadassah Faculty of Medicine, School of Public Health and Community Medicine, Jerusalem, Israel

**Keywords:** *Mycobacterium tuberculosis* complex (MTBC), next generation sequencing (NGS), whole genome sequencing (WGS), Israel, migrants, multidrug resistant *M.tb* (MDR-TB), extensively drug-resistant *M.tb* (XDR-TB)

## Abstract

According to World Health Organization WHO, Tuberculosis (TB) is the second cause of death from infectious disease worldwide. During 2021, 10.6 million people were infected with TB, and 1.6 million people died. TB is caused by pathogens belonging to the *Mycobacterium tuberculosis* complex (MTBC), mainly by *Mycobacterium tuberculosis* (*M.tb*). Members of this complex are acid-fast bacilli, which can cause intrapulmonary and extra pulmonary TB, and can be divided into various lineages, based on genomic markers. The main public health threat comes from drug resistant *M.tb* strains, which are responsible for about 25% of TB death and treatment failure worldwide. Treating drug resistant TB patients significantly raises the costs of TB treatment. This study provides an overview of the demographic and drug susceptibility characteristics of newly diagnosed TB patients in Israel in 2021. The State of Israel has a very low level of TB endemicity and is at a pre-elimination phase. Notably, only 11.7% of the newly diagnosed TB patients were born in Israel. In this report, of the 154 new laboratory-confirmed TB patients, 66.7% had pulmonary TB, while 16% had extrapulmonary TB. Males accounted for 52% of the patients, with the most prevalent age group being 21-40. Most patients were citizens of Israel (53.9%), while 37.7% had no Israeli citizenship. Among non-citizens, there was a predominance of males and patients aged 21-40. The susceptibility profile showed a high resistance rate to streptomycin (18.2%) and to a lower extent to isoniazid (13.6%), pyrazinamide (8.4%), rifampicin (7.8%), and ethambutol (3.2%). Only 2 cases of XDR-TB and 10 MDR-TB strains were detected in Israel in 2021, with both XDR strains and 5 out of 10 MDR strains belonging to the Beijing lineage. Most of Beijing isolates were resistant to at least one tested drug. Genomic sequencing of 134 out of 156 strains and bioinformatics analysis using the MTBseq program and WHO mutation catalogue shows a good match with only 9 discrepancies between phenotypic and genotypic susceptibility profiles in first line drugs. The most common lineage is Delhi-Cas (23%) followed by the Beijing lineage (17%). Most patients from the Delhi-Cas lineage were born in Africa, while patients with Beijing isolates were born in different countries. Minimum spanning tree analysis identified 15 clusters. The study highlights the need for ongoing surveillance of TB using molecular and phenotypic tools to further decreasing the spreading level of the disease and develop effective treatment strategies.

## Introduction

Tuberculosis (TB) is the second leading cause of death from infection (following COVID-19) and one of the top ten causes of death in low-income countries. (https://www.who.int/news-room/fact-sheets/detail/the-top-10-causes-of-death), (Trajman et al., 2022). It is a communicable disease, which mostly affects the lungs, as well as lymph nodes, pleura, genitourinary tract, abdomen, skin, bones, and brain (Lee, 2015). The infectious dose is low and ranges from 1 to 5 bacteria, and the interaction between the host immune defense system and the bacteria determines the development of clinical TB, latent TB or elimination of infection (de Martino et al., 2019). According to the World Health Organization (WHO), in 2021, an estimated 10.6 million people fell ill with tuberculosis (TB) worldwide, a total of 1.6 million people died from TB including 187,000 people co-infected with HIV (https://www.who.int/news-room/fact-sheets/detail/tuberculosis). COVID-19 pandemic led to a decrease in reported newly diagnosed TB cases, while causing an elevated TB death at the same time (https://www.who.int/publications/i/item/9789240037021).

Tuberculosis is caused by members of the *Mycobacterium tuberculosis* complex (MTBC), comprised of acid-fast bacilli pathogens (Gagneux, 2018). Among them, the common human pathogens are *Mycobacterium tuberculosis* (TB)*, Mycobacterium africanum (M. africanum)* and *Mycobacterium bovis* BCG (Kanabalan et al., 2021).

The major threat of TB arises from drug resistant TB (DR-TB) cases, which are more difficult to treat and constitutes a significant economic burden (https://www.who.int/activities/tackling-the-drug-resistant-tb-crisis). Multidrug resistant TB (MDR-TB) are resistant to the two most potent anti TB drugs: rifampicin and isoniazid. Another category is Extensively Drug- Resistant *M.tb* (XDR-TB) which are MDR-TB resistant also to a fluoroquinolone/s, and bedaquiline or linezolid (or both) (https://www.cdc.gov/tb/topic/drtb/default.htm). According to WHO 2021 report, 132,222 cases of MDR/RR-TB (rifampicin resistant) and 25,681 cases of pre-XDR-TB (MDR resistant to fluoroquinolone) & XDR-TB were detected during 2020 worldwide (https://www.who.int/publications/i/item/9789240037021).

Since 1996 there is an ongoing monitoring of new laboratory diagnosed TB cases in Israel, as part of the national program to eliminate TB (Chemtob et al., 2003). During the last 5 years there have been between 130-230 new cases each year with low TB mortality rate, and only few MDR-TB & XDR-TB cases. Israel encountered massive waves of immigrations. Most of TB patients are foreign born in TB endemic countries, including Jews with a right to get citizenship and migrant workers (Goldblatt et al., 2014; Shuldiner et al., 2014; Chemtob and Ogum, 2020).

Nowadays, Whole Genome Sequencing (WGS) is extensively used for identification, characterization and susceptibility testing of pathogens including MTBC. The *M.tb* catalogue of mutations, published by WHO in 2021, significantly contributed to the molecular identification of MTBC resistant strains. (https://www.who.int/publications/i/item/9789240028173). It is important to emphasize that WHO use WGS as reference standard for drug susceptibility testing for rifampicin, pyrazinamide and ethambutol, in combination with phenotypic drug susceptibility test (https://www.who.int/news/item/25-07-2023-who-issues-rapid-communication-on-use-of-targeted-next-generation-sequencing-for-diagnosis-of-drug-resistant-tuberculosis).

This is a first annual report of laboratory confirmed newly diagnosed TB cases in Israel which includes demographic, phenotypic and WGS data.

## Materials and methods

### Ethics statement

This study was approved by the National Helsinki Committee for Medical Experiments on Humans, of the Israeli Ministry of Health (# MOH-081-2021).

### Patient data

Demographic data and HIV status of patients were received from the Ministry of Health Department for Tuberculosis and AIDS and are managed according to WHO classification (https://www.gov.il/en/departments/units/tb_aids_unit/govil-landing-page). Patients information appears in **Supplementary Information Demography Table** **S3**.

### Data classification and identification of MTBC

This retrospective study included all laboratory confirmed TB cases in Israel in 2021. TB case was defined as a patient with disease due to MTBC bacteria, confirmed using culture from sputum, body fluid or tissue. All patients with suspected pulmonary TB in Israel are requested to provide sputum or other biological specimens for culture, while most suspected extrapulmonary cases, undergo tissue biopsy for laboratory testing (Mor et al., 2013). All cultures processed in Israel are sent to the National Mycobacterium Reference Laboratory (NMRL), in Tel Aviv, Israel. At the NMRL, smears from samples and TB cultures are stained using Ziehl-Neelsen staining and cultured on Lowenstein-Jensen (LJ) media using standard methods (https://ntrl.ntis.gov/NTRL/dashboard/searchResults/titleDetail/PB86216546.xhtml), and in addition cultured on BACTEC MGIT 960 (BD, Sparks, MD, USA) system. The species are identified using conventional biochemical methods (Marks, 1976; Collins et al., 1985; Laber and Burnham, 2023) and a commercially available strip DNA probe test (Hain Lifesciences, Nehren, Germany). *Mycobacteria* other than MTBC, as well as *M. bovis*, were excluded from this study (only *M. bovis* BCG were included).

### Drug susceptibility testing for MTBC strains

Drug susceptibility testing (DST) for first line drugs (isoniazid-INH, rifampicin-RIF, ethambutol-EMB, pyrazinamide-PZA and streptomycin-STR), was performed at the NMRL using BACTEC MGIT 960 assay. BACTEC MGIT 960 MDR strains and strains resistant to any 3 first line drugs are further tested using the resistance ratio method (**Supplementary Information Material Table** **S4**). Pyrazinamide (PZA) was tested using Mark’s stepped pH method (Marks, 1976; WHO, 2006). The following drugs were tested using resistance ratio method: ciprofloxacin (CIP), clarithromycin (CLA), capreomycin (CAP), cycloserine (CS), ethionamide (ETH), ofloxacin (OFX), clofazimine (CFZ) and amikacin (AMK). In this study, extensively drug resistant TB (XDR-TB) was defined using the previous definition: MDR-TB that is also resistant to any fluoroquinolone and to one or more of the injectable drugs AMK, CAP or kanamycin (KAN) (Kabahita et al., 2022).

Drugs threshold levels used to determine resistance are described in **Supplementary Information Material T** **able S4**. Importantly, for the purpose of the article, the categories Borderline and RR4 were redefined as Intermediate.

### Whole genome sequencing and bioinformatics

All the bacteriological culture and sequencing were done in the NMRL. 138 out of 156 strains were sequenced and analyzed, 18 contaminated cultures were not sequenced. Genomic DNA from inactivated bacterial cultures was extracted according to the manufacturer’s protocol using Maxwell RSC cultured cells DNA kit and Maxwell^®^ 16 System (Promega) (Cabibbe et al., 2018). DNA Paired-end libraries were prepared using the Illumina Nextera XT DNA Library Preparation Kit according to Illumina protocols. For sequencing, we utilized the Illumina MiSeq platform using a MiSeq Reagent Kit v2 (500-cycles) (catalogue MS-102-2003) or a MiSeq Reagent Kit v3 (600-cycle), (catalogue MS-102-3003).

Raw reads fastq files were quality analyzed by FastQC (https://www.bioinformatics.babraham.ac.uk/projects/fastqc/). Kraken2 taxonomic classification (Wood et al., 2019) was used to identify mixed cultures. MTBseq, a comprehensive pipeline for WGS analysis of MTBC isolates, was used as the main tool for MTBC analysis of lineage, identification of genomic variations and clusters analysis (Kohl et al., 2018). Lineages were identified as part of the MTBseq pipeline, based on the SNP scheme developed by Coll et al. (Coll et al., 2014). Genomic variations were compared to the WHO 2021 Catalogue of drug resistance associated mutations in MTBC, and used to identify resistant strains to antibiotics (https://www.who.int/publications/i/item/9789240028173).

In order to perform a comparison between phenotypic and genotypic susceptibility test results, we defined the following categories in the WHO catalogue final confidence grading as resistant: ‘associated with resistance’ and ‘associated with resistance-interim’, as well as mutations not in the catalogue which nevertheless comply with expert rules used for the final confidence grading. Similarly, the phenotypic susceptibility results were categorized into sensitive (including borderline) and resistance (including RR4).

### Statistical analysis

All the calculations were done using Microsoft Excel software. Sensitivity and specificity of WGS resistance prediction were obtained using R v4.2.1 (https://www.r-project.org/).

### Data availability

Illumina sequences are available to the public at the NCBI (BioProject PRJNA957554).

## Results

In 2021, 154 new laboratory confirmed TB patients were reported in Israel, among them 66.7% with intrapulmonary TB (sputum, bronchial wash and stomach fluid) and 16% with extrapulmonary TB (urine, blood, CSF, lymphatic gland, peritoneal fluid, pleural fluid, synovial fluid, wound) (**Supplementary Information Table** **S1**, **Supplementary Information Figure 1**). Two of the patients had 2 different types of positive samples for each patient, summing up to a total of 156 isolates. 93% of the patients were infected with *M. tuberculosis*, 6% with BCG and only 1% with *M. africanum*. 7.1% (11) of the patients are HIV positives.

**Figure 1:**
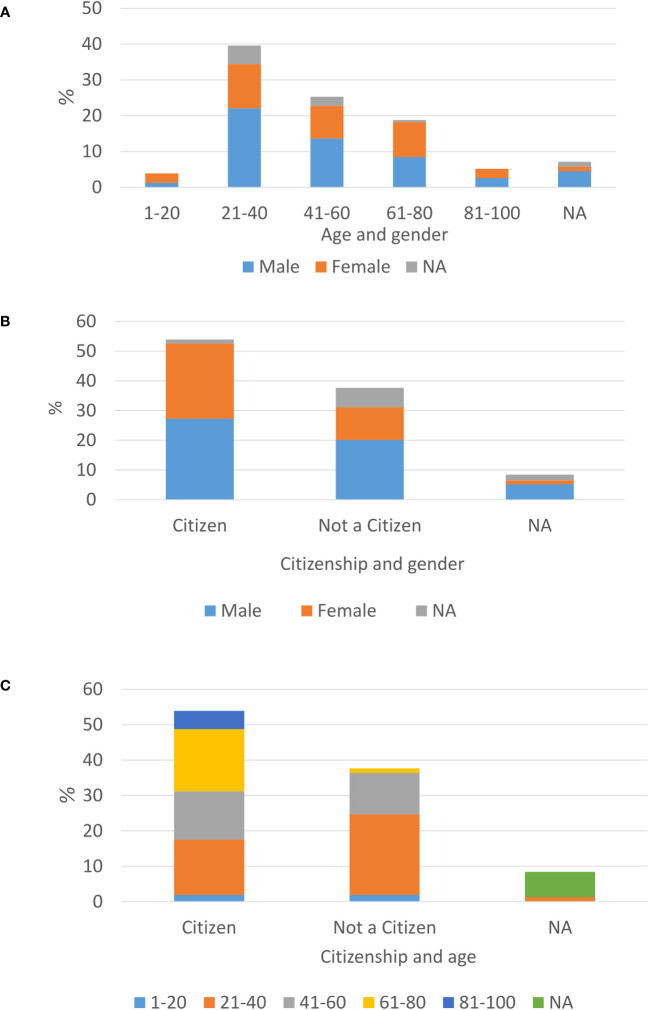
Demographic characterizations of newly laboratory identified TB patient is Israel, year 2021: Age distribution among new TB diagnosed males and females **(A)**, Citizenship status among TB males and females **(B)**, age distribution among citizens vs. non-citizens **(C)**.

Most of the reported patients were males (52% males *vs.* 38% females), and the most prevalent age was 21-40 (39.6%). There is a clear bias in age distribution among genders, toward males in the age of 21-40 (22%) (**Figure 1A**). During 2021, most of the newly diagnosed TB patients were Israeli citizens (53.9%), vs. 37.7% not Israeli citizens. Among patients without an Israeli citizenship, there is a majority of males (31 males vs. 17 females out of 58, 53.4%, 29.3%, respectively) (**Figure 1B**). Among Israeli citizens, the distribution is approximately even (42 males vs. 39 females, out of 83, 50.6%, 47%, respectively). Additionally, among non-Israeli citizens, there is a majority of patients aged 21-40 (35 out of 58, 60.3%) (**Figure 1C**). Among new patients up to 40 years old males constitute most of the cases, while in older ages this trend decreases and the number of cases is similar among males and females (**Figure 1A**).

**Figure 2:**
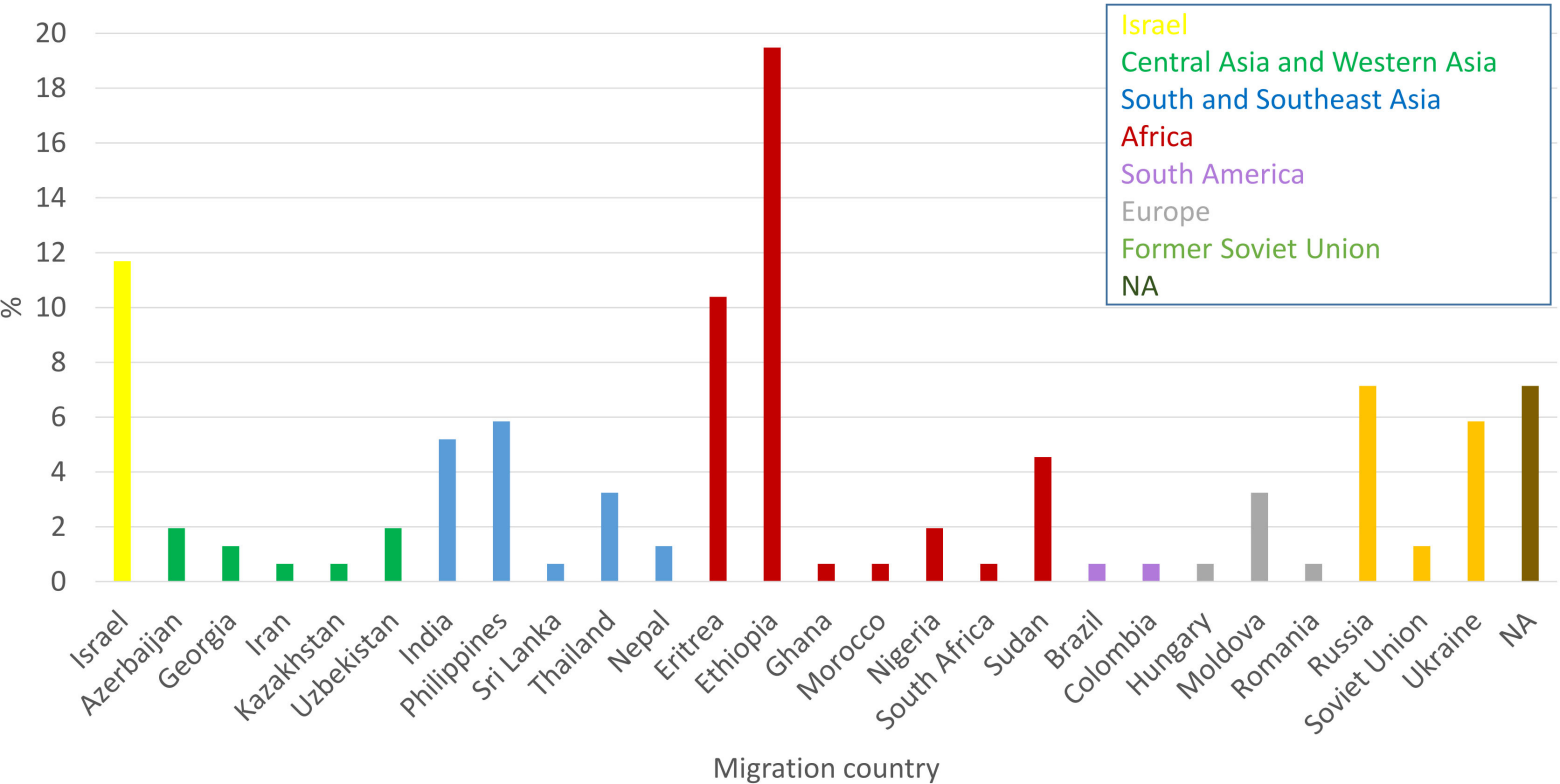
Country of birth of newly diagnose TB patients in Israel in 2021: Cases from different countries were colored as indicated inset.

Only 11.7% of the newly diagnosed TB patients in 2021 were born in Israel, while 38.3% were born in Africa, 16.23% in South and Southeast Asia, 14.3% in Former Soviet Union (FSU, 20 out of 22 were from Russia and Ukraine, and for 2 patients there is no specific country information), 6.5% in Central Asia and Western Asia, 4.5% in Europe and 1.3% in South America (**Figure 2**).

**Figure 3:**
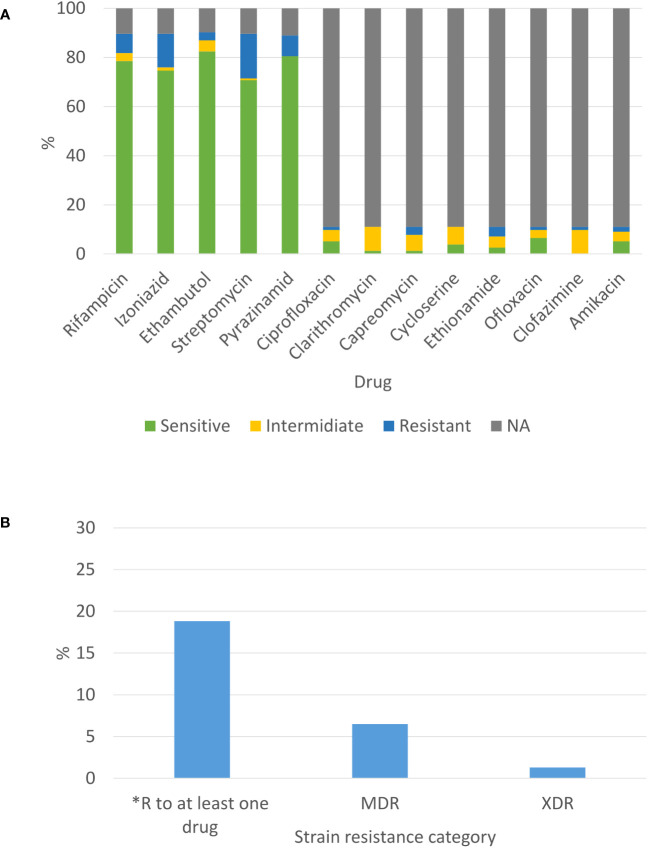
Phenotypic susceptibility profile of newly diagnosed TB patients in Israel in 2021: **(A)** Susceptibility to selected drugs from first and second line and **(B)** Drug resistance combinations. *excluding MDR-TB isolates.

For most of the isolates, the susceptibility profile is composed of the first line drugs. Only a small portion of strains were tested for the second line drugs (according to the criteria mentioned in the material and method section). The highest resistant rate among these drugs was 18.2% for streptomycin. Next, 13.6% of tested strains were resistant to isoniazid, 8.4% to pyrazinamide, 7.8% to rifampicin and 3.2% to ethambutol (**Figure 3**). Notably, because we do not test for susceptibility to Bedaquiline and Linezolid yet, we refer to the previous WHO definition of categorization to MDR and XDR which were modified in 2021(https://www.who.int/news/item/27-01-2021-who-announces-updated-definitions-of-extensively-drug-resistant-tuberculosis). Two new XDR-TB strains and 10 MDR-TB strains were detected in 2021 (1.3% XDR and 6.5% MDR of all 2021 strains). Both XDR-TB strains do not belong to the same genomic cluster (we found 157 SNP differences between them) (**Figure 3A, B**). Both XDR-TB strains and 5 out of 10 MDR-TB strains belong to Beijing lineage. Additionally, 2 out of the 11 isolates of HIV positive patients are resistant to rifampicin, 2 to ethambutol, 4 to streptomycin, 2 to pyrazinamide and 4 to isoniazid. Finally, 2 out of 11 HIV positive strains are MDR.

Genomes of 134 out of 156 strains were fully sequenced and drug susceptibility was predicated using the WHO catalogue of mutations in *Mycobacterium tuberculosis* complex and their association with drug resistance (https://www.who.int/publications/i/item/9789240028173). We compared the phenotypic and genotypic susceptibility profiles of these strains and found excellent agreement between the 2 methods, especially for first line drugs (**Table 1**). The discrepancies between these 2 methods were mainly scattered among the different second line drugs (**Table 2**). All the information regarding the specific mutations that confer resistant to first line anti TB drugs in the tested strains can be found in the **Supplementary Information Table S2**.

**Table 1:** The sensitivity and specificity of genomic-based drug resistance prediction, compared to phenotypic DST.

	INH	STR	EMB	RIF	PZA	AMK	CAP	CFZ	ETH	FQs
Prediction sensitivity	1.00 (n=18)	0.85 (n=26)	1.00 (n=8)	0.75 (n=12)	0.91 (n=11)	0.80 (n=5)	0.50 (n=6)	0.00 (n=7)	0.71 (n=7)	0.75 (n=4)
Prediction specificity	1.00 (n=109)	0.98 (n=101)	0.99 (n=120)	1.00 (n=115)	1.00 (n=115)	1.00 (n=8)	1.00 (n=7)	1.00 (n=6)	1.00 (n=6)	1.00 (n=9)

The numbers in parentheses indicate the total number of relevant strains, namely the total number of phenotypically resistant strains for the sensitivity of prediction, and the total number of drug-sensitive strains for the specificity of prediction. INH, isoniazid; STR, streptomycin; EMB, ethambuto; RIF, rifampicin; PZA, pyrazinamide; AMK, amikacin; CAP, capreomycin; CFZ, clofazimine; ETH, ethionamide; FQs, fluoroquinilones.

**Table 2:** Comparing the phenotypic and genotypic (whole genome sequence) susceptibility testing results: it can be seen that the differences are minor.

	Drug	Case #	Locus ID	Gene Name	Mutation	WHO confidence grade	WHO PPV SOLO
False positive – genotype was resistant and phenotype was sensitive	**Streptomycin**	A	Rv3919c	*gid*	L35frameshift	1) Assoc w R	58%
		B	Rv3919c	*gid*	R39frameshift	2) Assoc w R - Interim	0%
	**Ethambutol**	C	Rv3795	*embB*	M306I	1) Assoc w R	53%
False negative – genotype was sensitive and phenotype was resistant	**Rifampicin**	D, E,F					
	**Pyrazinamide**	G					
	**Streptomycin**	H, I, J, K					
	**Amikacin**	L					
	**Capreomycin**	L, M, N					
	**Clofazimine**	C, F, L, M, O, P, Q					
	**Ethionamide**	P, R					
	**Fluoroquinolones**	P					

Specific cases of the disagreement detailed in the table.

**Figure 4:**
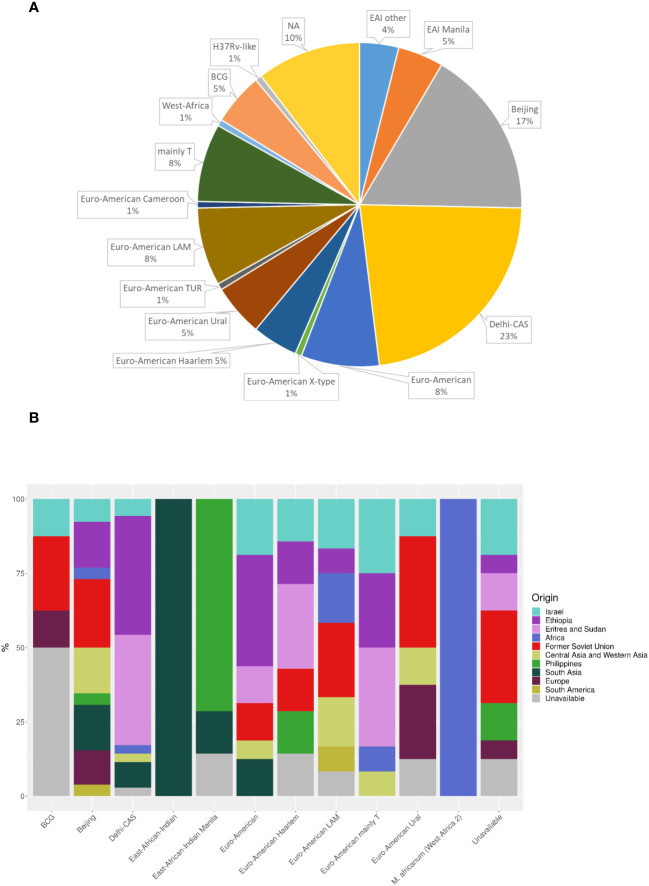
MTBC lineage of newly diagnosed TB patients **(A)**, and place of birth distribution among the lineages **(B)** in Israel in 2021. NA - the isolates were not sequenced. (n=154). Dehli-CAS, the major lineage in Israel consists mainly of patients from Africa. In contrast, patients infected with Beijing lineage, were born in many countries and regions including Israel, Africa, FSU, Central Asia and Western Asia, South America, Philippines, South Asia and Europe.

**Figure 5:**
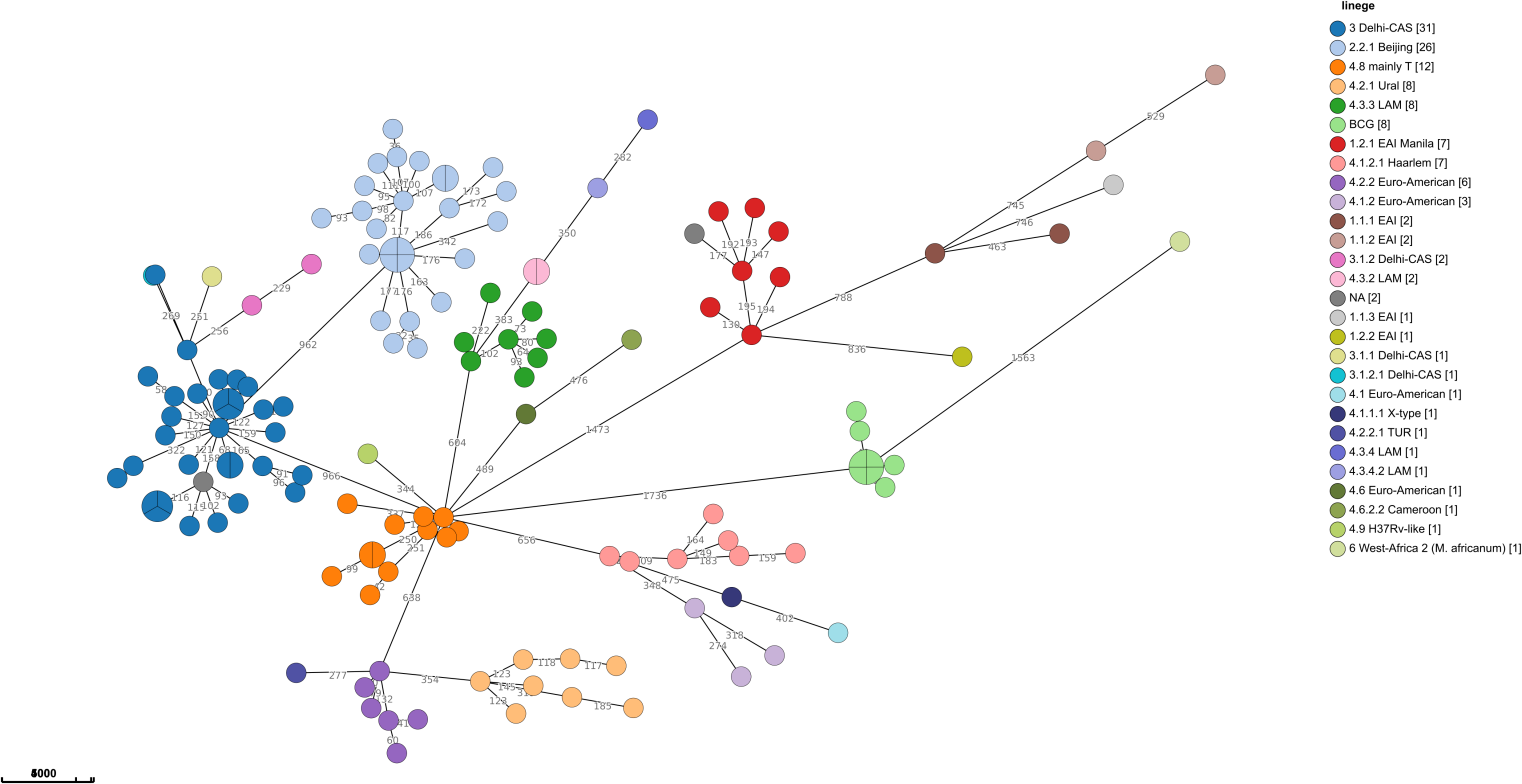
Minimum spanning tree of MTBC in Israel in 2021: The size of the circles is proportional to the number of isolates in the cluster. The color of the circles is according to lineages, the length of the lines connecting the circles is proportional to the number of SNPs. The numbers along the line connecting the circles indicate the SNPs numbers. A cluster of cases is defined when the distance between all isolates in the cluster is equal to or less than 12 SNPs, (n=138).

Sequencing analysis showed that Israel has a high variety of lineages (16 lineages in 2021), the most common ones are Delhi-Cas (22.7%) and Beijing (16.9%) (**Figure 4A**). **Figure 4B** shows the association between TB lineage and the country of patient’s birth. It can be seen from **Table 3** that Beijing is a highly “resistant” lineage. It contains isolates resistant to each one of the 5 first line drugs (Out of 26 strains, 7 shows resistant to Rifampicin, 9 to isoniazid, 4 to ethambutol, 18 to streptomycin and 4 to pyrazinamide). In contrast, Delhi-CAS, the major lineage in Israel in 2021, consists of only 1 strain resistant to isoniazid, out of 35 strains, and no representation of resistant strains to other antibiotics. BCG is intrinsically resistant to pyrazinamide (Jureen et al., 2008). Indeed, 7 out of 8 BCG isolates are resistant to pyrazinamide. The NMRL routinely compares newly sequenced isolates to all previous isolates in order to identify clusters. Clusters are defined by a difference of 12 SNPs or less. All clusters identified by the NMRL are reported to the related District Health Bureau and to the Tuberculosis & AIDS department of the ministry of health, which are responsible to perform epidemiological investigation. Although strains from the same lineage co-localize adjacently on the tree, it is nevertheless apparent that lineage information alone is not enough to understand epidemiological causality. In 2021 we identified 15 clusters (**Figure 5**).

**Table 3:** Phenotypic drug resistance profiling of MTBC by lineage classification.

Lineage	#strains	Pyrazinamide	Streptomycin	Ethambutol	Izoniazid	Rifampicin
Delhi-CAS	**35**	0	0	0	1	0
Beijing	**26**	4	18	4	9	7
Euro-American	**16**	0	1	0	1	0
NA	**16**	2	3	1	3	3
Euro-American LAM	**12**	0	3	0	2	1
Euro-American mainly T	**12**	0	0	0	0	0
Euro-American Ural	**8**	0	1	0	1	1
BCG	**8**	7	0	0	0	0
Euro-American Haarlem	**7**	0	2	0	0	0
East-African-Indian Manila	**7**	0	0	0	0	0
East-African-Indian	**6**	0	0	0	3	0
M. africanum (West-Africa 2)	**1**	0	0	0	1	0
Total	**154**					

NA- isolate not sequenced.

## Discussion

This study provides the most comprehensive analysis to date regarding tuberculosis cases in the State of Israel. It encompasses all new cases identified in 2021 and it also includes a detailed description of the demographic parameters as well as a drug resistance analysis, utilizing both phenotypic and genomic methods. Finally, it includes a cluster analysis of the genomic data.

According to the WHO and the international literature, TB is more prevalent in males than in females, and the bias is gradually increasing in patients older than 25 years old (https://www.who.int/teams/global-tuberculosis-programme/tb-reports/global-tuberculosis-report-2022; (Marcoa et al., 2018). Although the incidence of tuberculosis in Israel is higher in men than in women (as shown in **Figure 1**), the major gender bias appears in patients aged 21-40 (**Figure 1**). The gender ratio among Israeli citizens is approximately one, while non-Israeli citizen patients are predominantly males. Further analysis of the data by age group (as depicted in **Figure 1C**) reveals that non-Israeli citizen patients (**Supplementary Information Demography Table** **S3**) are primarily between the ages of 21-40. We speculate that some of them may be migrant workers. **Figure 2**, shows that the main regions of birth of TB patients in 2021 are Africa, the FSU, South and Southeast Asia. These are TB endemic regions, which emphasizes the significant role of migrant among TB patients in Israel (https://www.who.int/teams/global-tuberculosis-programme/tb-reports/global-tuberculosis-report-2022/tb-disease-burden/2-1-tb-incidence).

Since its establishment, Israel has become a melting pot for immigrants from various parts of the world, including the FSU, Africa, and Asia. In 2021 alone, 34.1 thousand new immigrants arrived to Israel; of them 25.5 thousand immigrated according to the Israeli Law of Return. Additionally, at the end of 2021, around 123 thousand foreign-nationals holding a valid work visa resided in Israel. Notably, legal immigrant workers in Israel are screened for TB infections in their origin counties using chest x-ray. The majority of incoming foreign workers in 2021 came from Thailand (22.4%), the Philippines (21.9%), India (12.5%), China (10.3%), and Sri Lanka (3.8%). In terms of illegal immigration, at the end of 2021, approximately 28.1 thousand illegal residents remained in Israel, with the majority being from Eritrea (74%) and Sudan (18%) (https://www.cbs.gov.il/EN/pages/default.aspx). Furthermore, many Israelis temporarily leave the country for various reasons, such as work, study, and travel. As a country of significant immigration, the number of new TB cases in Israel, shows correlation to the large immigration waves. For example, the large immigration from FSU during the 1990th is in correlation with the significant peak of newly laboratory confirmed TB cases in these years (https://www.gov.il/he/departments/publications/reports/lab-ta2021).

The 2 most prevalent TB lineage in Israel are Cas and Beijing. Goldblatt et al., 2014 (Goldblatt et al., 2014) reviewed the epidemiology of tuberculosis in Israel during 2008-2010, they present a close connection between the genetic lineages common in the countries of origin of immigrants, and the lineages isolated from these patients in Israel. Furthermore, epidemiological investigations did not show transmission between immigrants and Israeli born patients. The major TB lineages in that work, determined by spoligotyping, were Central Asian (CAS) (20%), Beijing (15%) and T (23%) (Goldblatt et al., 2014). In contrast to Goldblatt et al., in our study the lineage was determined from the genomic sequence and not by spoligotyping. Comparison between the results of traditional spoligotyping and genome base lineage determination (MTBseq, SNP based, (Coll et al., 2014)) showed a good correlation, especially in major lineages like Cas, Bovis, Beijing and Haarlam, (data not shown), allowing comparison of results obtained by either method. Discrepancies appear mainly between spoligotype T1 that is defined by the SNP scheme as different Euro-American lineages. While the results for the major lineages Cas and Beijing are very similar in both studies (Cas 20% vs. 23%, Beijing 15% vs. 17%, Goldblatt vs. this study), the situation is completely different regarding the Haarlem lineage, which was about 12% of all isolates in 2008-2010 and only 5% in 2021 (**Figure 4A**). A very wide variety of other genetic lineages were detected in 2021 (**Figure 4A**) reflecting the origin of TB in Israel from many different countries.

Another major difference between Goldblatt et al., and the current study is in the origin of Beijing isolates, which in the previous study was mainly the FSU (66%) while in our study they originated from a wide range of regions (**Figure 4B**), probably reflecting the changes in Israel demography over the years and the global spread of this lineage (Merker et al., 2015).

In this study, like many others, the Beijing lineage leads in resistance to anti-tuberculosis drugs (**Table 3**), (European Concerted Action on New Generation Genetic, M., Techniques for the, E., and Control of, T., 2006; Hakamata et al., 2020; Liu et al., 2020) (Merker et al., 2015). Most of Beijing strains in 2021 are resistant to at least 1 tested drug, and both XDR and 5 MDR TB strains are from Beijing lineage (**Table 3**).

In another comprehensive study comprised of 4,102 TB isolates belonging to the Cas lineage, from many countries, the prevalence of MDR-TB and XDR-TB was found to be 30.63% and 1.03%, respectively (Couvin et al., 2019). Surprisingly, our data showed relatively low anti TB resistant among Cas lineage isolates, with only 1 isolate resistant to isoniazid (**Table 3**). This finding stands in stark contrast to the high rates of drug resistance observed among other TB lineages. One possible explanation for this discrepancy is that many of the Cas isolates in Israel originates from immigrants born in African countries, mainly Ethiopia, Eritrea and Sudan (EES), **Figure 4B**, (Goldblatt et al., 2014), with limited access to medical services. In a recent meta-analysis from Ethiopia, the prevalence of any anti-TB drug resistance was 14.25% (Reta et al., 2022). In contrast, another meta-analysis from Sudan, shows 47.0% resistance to any anti-TB drugs (Hajissa et al., 2021). In a recent study from Eritrea, Cas lineage accounts for about 62% of all isolates and the maximal resistance detected for streptomycin and isoniazid was 4.7% each (Mesfin et al., 2021). In conclusion, there is a low level of drug resistance stains in most African countries (except Sudan) from which there are TB patients in Israel. These findings are consistent with our hypothesis. It should be noted that in the abovementioned studies, the TB resistance data is a summary of all isolates without sub-categorizing to TB lineages, which makes lineage-specific comparison to our study more difficult.

BCG account for 5% of MTBC isolates (**Figure 4A**). BCG positive results are commonly attributed to BCG vaccine and treatment for urinary bladder cancer. BCG administered to the bladder, can find its way out of the bladder, a rare complications of the treatment (Lussier et al., 1999; Green et al., 2019). We have only partial medical information regarding some of these patients. Two out of 9 BCG positive patients received BCG vaccine. The origin of these samples is bronchial wash and biopsy; 3 out of 9 BCG patients were treated with BCG for urinary bladder cancer. The origin of these samples is urine in 2 of the cases and abscess in the testicles in the third one.

Pulmonary TB, is the most common type of TB worldwide. It involves the lungs, and the specimen type tested are lung secretions obtained in various ways, such as sputum, laryngeal swabs, gastric lavage, bronchial wash, etc. As extrapulmonary TB affects organs other than the lungs, samples are taken from different types of the body fluids (spinal, pleural, synovial, bone marrow, blood, urine) and tissues (https://www.ncbi.nlm.nih.gov/books/NBK138741/), (https://books.google.co.il/books/about/Public_Health_Mycobacteriology.html?id=lKfnh5R4_CEC&redir_esc=y). Extrapulmonary TB rate is about 15-20% among TB cases, and immunosuppression and HIV being among the main risk factors (Sharma and Mohan, 2004; Elango et al., 2022). Israel falls in the statistics with 16% extrapulmonary TB in 2021 (**Supplementary Information Table** **S1**, **Supplementary Information Figure 1**) as well as during 2008-2020 as reported by Goldblatt et al. (Goldblatt et al., 2014).

Drug resistant TB is a public health threat, especially MDR and XDR cases. Globally, the number of rifampicin resistant, MDR, Pre-XDR or XDR cases was 157,903 in 2020 (Pre-XDR is a strain of MDR TB resistant to any fluoroquinolone) (https://www.who.int/publications/i/item/9789240037021). WHO data emphasize that there is a clear reduction in the total number of TB patients confirmed by laboratory, as well as a decrease in confirmed resistant TB cases in 2020, compared to previous years. This change is attributed to the COVID-19 pandemic (Trajman et al., 2022). The same trend was observed in Israel (data not shown). Our data show that in Israel, the proportion of MDR and XDR in relatively low (7.7%, 12 out of 154, **Figure 3B**), Goldblatt et al, identified 29 (4%) MDR-TB isolates during 2008-2010 (Goldblatt et al., 2014).

In this study, WGS was analyzed using MTBseq pipeline in combination with the WHO mutation catalogue. For the best of our knowledge, this is the first publication describing such an approach. As shown in **Table 1**, the overall specificity and sensitivity of our WGS pipeline for first line drugs are excellent. For second line drugs, the overall performance was lower (**Table 2**). Only 3 isolates had mutations known to be associated with resistance, yet tested phenotypically sensitive (false positive). It is difficult to explain these cases. On the contrary, 15 isolates that were predicted to be sensitive based on their genomic sequence, yet proved resistant by the phenotypic DST (false negative). This could be explained, for example, by mutations which are not yet known. Since only strains resistant to first line drugs are tested for second line drugs, only a few isolates were tested phenotypically for susceptibility for second line drugs, thus a reliable comparison is not possible. Notably, lesser prediction for second line drugs was observed by others as well (Ngo and Teo, 2019). The main limitation is lack of global knowledge regarding mutations that confer resistance to second line drugs. Our laboratory collaborates with the WHO catalogue team, and shares phenotypic and genotypic data in order to gradually expand this database.

The routine use of Nucleic Acid Amplification Test (NAAT) allows a short TAT (turn-around time, the time period between the arrival of a specimen to the laboratory and the report of the result). On the other hand, NAAT kits do not cover all known resistance mutations, especially resistance to second line drugs, which is very limited nowadays. In the future, we expect to have a better knowledge of the genomic determinants of resistance. In recent years, understanding the connection between genomic and phenotypic resistant is gradually improving, as well as the sequencing technologies and bioinformatics pipelines. In this context, WGS are favorable to NAAT, as it provides more detailed and broad information about the resistome in a single assay. WGS also provides high resolution epidemiological links between isolates.

In conclusion, this study provides comprehensive analysis of all new laboratory confirmed TB cases notified in Israel in 2021, and includes demographic, drug resistance and genomic analysis. The data show that TB is more prevalent in males than females, with a significant bias in patients aged 21-40 who are primarily non-citizens. The major TB lineages in Israel are Cas and Beijing, with Beijing lineage leading in resistance to anti-tuberculosis drugs. The study also highlights the significant number of immigrants among new TB cases in Israel. This study emphasizes the benefits of using WGS in characterizing *M. tb* strains. The results obtained in this study are in line with previous studies and contribute to grasp the epidemiological characteristics of laboratory confirmed tuberculosis in Israel.

### The study has several limitations

Small sample size: The study has a small number of cases (154), with only 134 genomes sequenced. This small number of patients may limit the statistical power of the study.

Missing demographic data: Demographic data such as age, gender, and ethnicity as well as clinical data and medical history are missing for some patients included in the study.

Cross-sectional design: The study uses a cross-sectional design (all laboratory confirmed, new TB patients in 2021). Longitudinal studies that follow TB over years may provide more information about demographic, lineage, clusters and resistance dynamics.

## Data availability statement

The datasets presented in this study can be found in online repositories. The names of the repository/repositories and accession number(s) can be found below: NCBI, Bioproject #: PRJNA957554.

## Ethics statement

The studies involving humans were approved by the National Helsinki Committee for Medical Experiments on Humans, of the Israeli Ministry of Health (# MOH-081-2021). The studies were conducted in accordance with the local legislation and institutional requirements. The human samples used in this study were acquired from a by- product of routine care or industry. Written informed consent for participation was not required from the participants or the participants’ legal guardians/next of kin in accordance with the national legislation and institutional requirements.

## Author contributions

YL, MR, and IN: conceived the manuscript concept, collected and analyzed the data, and wrote the manuscript. PH, YB, MV, GB-G, TZ, MH, GV, IK: conducted experiments and analyzed data. HS: project supervision and critical reading of the manuscript. DC: DC is in charge of the MoH Department of TB and AIDS, which is managing the National TB registry. This department collected, managed and is responsible for the quality of the data. Those data were provided to the National TB Lab. ZD, DC: critical reading of the manuscript. ER: project supervision, conceived, designed the experiments, and critical reading of the manuscript. All authors contributed to the article and approved the submitted version.
