# Supplementary figures and images for: Genomic, phenotypic and demographic characterization of Mycobacterium tuberculosis in Israel in 2021
Source: Front Cell Infect Microbiol. 2023 Oct 18;13:1196904. doi: 10.3389/fcimb.2023.1196904 (PMC10622789; doi:10.3389/fcimb.2023.1196904)

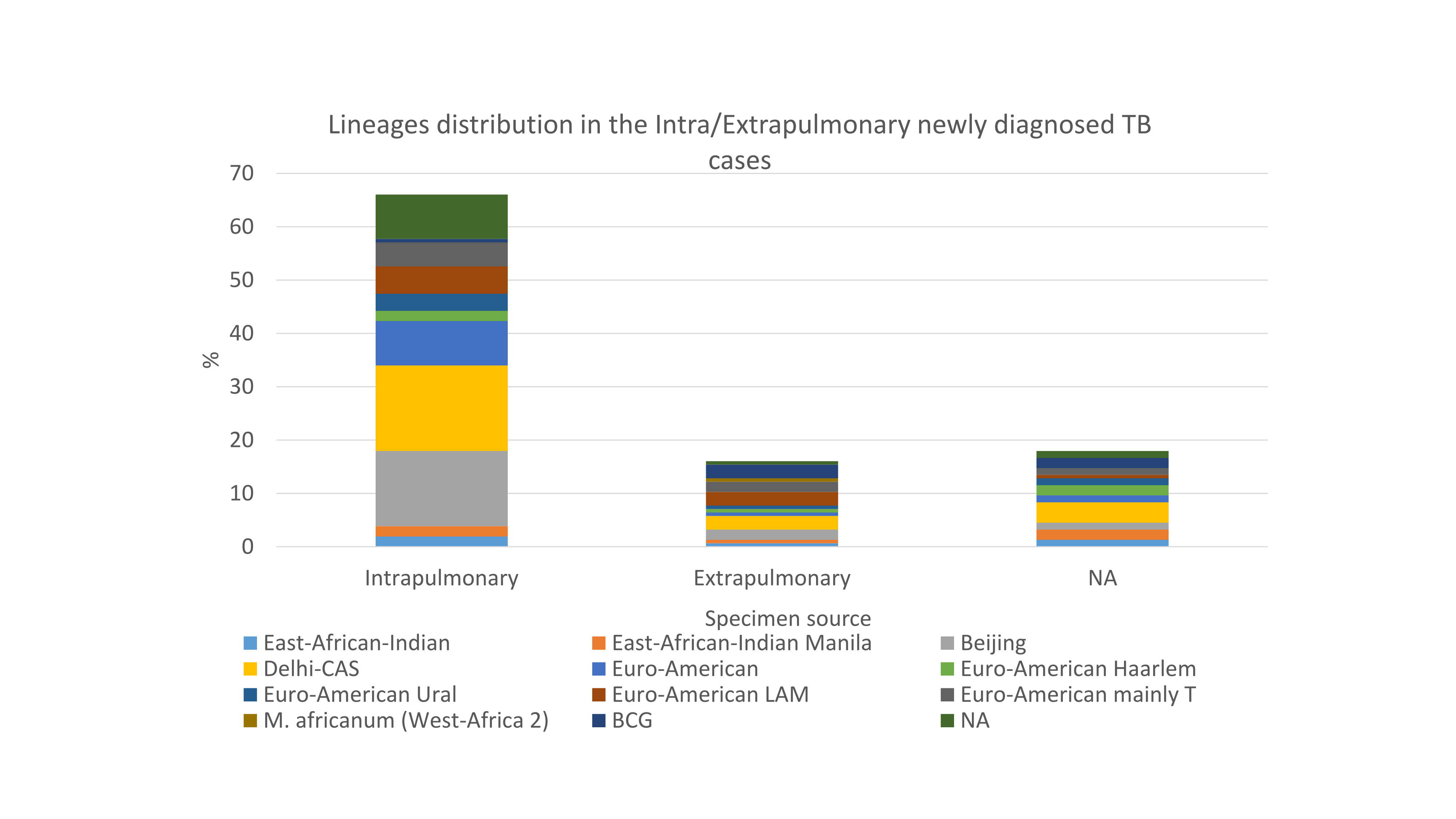

Supplement: Supplementary file 1 [file Image_1.tif]
